# The Effects of Run-of-River Hydroelectric Power Schemes on Fish Community Composition in Temperate Streams and Rivers

**DOI:** 10.1371/journal.pone.0154271

**Published:** 2016-05-18

**Authors:** Gary S. Bilotta, Niall G. Burnside, Jeremy C. Gray, Harriet G. Orr

**Affiliations:** 1 Aquatic Research Centre, School of Environment and Technology, University of Brighton, Brighton, United Kingdom; 2 Department of Ecology and Evolutionary Biology, University of Toronto, Toronto, Ontario, Canada; 3 Environment Agency, Bristol, United Kingdom; University of Delhi, INDIA

## Abstract

The potential environmental impacts of large-scale storage hydroelectric power (HEP) schemes have been well-documented in the literature. In Europe, awareness of these potential impacts and limited opportunities for politically-acceptable medium- to large-scale schemes, have caused attention to focus on smaller-scale HEP schemes, particularly run-of-river (ROR) schemes, to contribute to meeting renewable energy targets. Run-of-river HEP schemes are often presumed to be less environmentally damaging than large-scale storage HEP schemes. However, there is currently a lack of peer-reviewed studies on their physical and ecological impact. The aim of this article was to investigate the effects of ROR HEP schemes on communities of fish in temperate streams and rivers, using a Before-After, Control-Impact (BACI) study design. The study makes use of routine environmental surveillance data collected as part of long-term national and international monitoring programmes at 23 systematically-selected ROR HEP schemes and 23 systematically-selected paired control sites. Six area-normalised metrics of fish community composition were analysed using a linear mixed effects model (number of species, number of fish, number of Atlantic salmon—*Salmo salar*, number of >1 year old Atlantic salmon, number of brown trout—*Salmo trutta*, and number of >1 year old brown trout). The analyses showed that there was a statistically significant effect (p<0.05) of ROR HEP construction and operation on the number of species. However, no statistically significant effects were detected on the other five metrics of community composition. The implications of these findings are discussed in this article and recommendations are made for best-practice study design for future fish community impact studies.

## Introduction

According to forecasts by the International Energy Association [1], electricity generation from renewables could nearly triple between 2010 and 2035, reaching 31% of total generation, with half of this from hydroelectric power. The contemporary methods used to generate hydroelectric power (HEP) are often site-specific and tailor-made to local conditions, but by far the greatest proportion of global HEP comes from large-scale storage-type schemes whereby rivers are dammed to create reservoirs [2]. The environmental effects of such large-scale storage schemes have been well-documented in the literature [3–5]. It is now recognised that the dam structures of large storage-type schemes can create obstacles for the movement of migratory fish species. They may also reduce access to spawning grounds and nursery areas, leading to a decrease in migratory fish populations and fragmentation of non-migratory fish populations [6]. Migratory and non-migratory fish can also be affected directly through injury or mortality resulting from contact with intake screens or turbine blades [7,8]. Storage-type schemes can also significantly modify the downstream flow regime (i.e., the magnitude and timing of discharge and hence water levels), and may also alter water temperature and quality [6]. The change in the annual flow pattern, combined with changes to sediment transport caused by water storage and controlled-release, can significantly affect natural aquatic and terrestrial habitats in the river and along the shoreline and floodplain. In some regions the storage of water can be associated with high evaporative losses, resulting in high lifecycle water footprints compared to other sources of electricity [9].

In Europe, it is the knowledge of these potential impacts, together with the recognition that most opportunities for economically-profitable and politically-acceptable medium-to large-scale schemes have already been developed [10–11], that has caused attention to turn to smaller-scale HEP opportunities, particularly run-of-river schemes, to help meet renewable energy targets [12]. The low greenhouse gas emissions from ROR HEP schemes (median lifecycle emissions of 4 grams of CO_2_ equivalent per kW hour of electricity generated) relative to other sources of electricity (including that from coal, 1001 gCO_2_e kWh^-1^; natural gas, 469 gCO_2_e kWh^-1^; solar PV, 46 gCO_2_e kWh^-1^; nuclear, 16 gCO_2_e kWh^-1^; and wind, 12 gCO_2_e kWh^-1^), certainly make ROR HEP an attractive option from the perspective of reducing the climatic impacts from electricity generation [6,13].

Run-of-river (ROR) schemes are HEP schemes that operate without water storage, using the flow within a river channel. Channel obstructions, typically weirs, are normally used to regulate water levels, allowing a proportion of flow to be diverted down a secondary channel to a turbine before it is returned to the main channel further downstream [2,12]. As such the life-cycle water footprint per kW hour of electricity generated has been found to be close to zero for ROR HEP schemes [14]. Run-of-river HEP schemes are also presumed to be less environmentally damaging than storage HEP schemes because they are normally built on, or make use of, existing weirs rather than involving the construction of large dams [11,15]. Some modern turbine types used in ROR HEP schemes are also designed to allow fish to pass through the system unharmed if the fish do pass through the intake screens. However, as highlighted in a recent literature review by Anderson et al., [12], there is currently a lack of peer-reviewed studies on the physical and ecological impact of these types of schemes. There is, therefore, a need to improve current understanding of the potential impacts of such schemes. This is particularly pertinent in the UK and Europe, where there has been a recent surge in HEP development [16,17], stimulated by financial subsidies from European and national renewable energy policies and legislative targets, but also a legislative requirement for all waterbodies to reach ‘good ecological status’ under the EU Water Framework Directive (*2000/60/EC*).

Previous research in this field has been constrained by the absence of long-term standardised data and weak study design; mainly relying on post-construction dynamics and spatial comparison with upstream and/or downstream reference reaches, which limits the conclusions that can be drawn [12,18]. The aim of this article is to investigate the effects of ROR HEP schemes on communities of fish in temperate streams and rivers, using a Before-After, Control-Impact (BACI) study design [19,20], that makes use of long-term routine environmental surveillance data collected according to standardised methods as part of national and international monitoring programmes.

## Materials and Methods

### Systematic search for operational ROR HEP schemes

The ROR HEP schemes included in this study were selected from a systematic search for HEP schemes, operating in England and Wales, which have meta-data available on their precise location, design, and dates of installation. There is no list that is publically-available for the UK that contains all of this information. However, each country’s respective regulatory authority (Environment Agency; Natural Resources Wales) collects information on proposed hydropower schemes when the developers apply for licences to abstract and/or transfer water from a river. This information provides a useful starting point for systematically identifying operational HEP schemes, but the limitations of this information are that not all schemes that are licenced get built, and the schemes that are built are not always constructed to the specifications detailed in the proposal. Furthermore, the information does not include a date of installation/commissioning, which is required to conduct a before-and-after analysis. Therefore independent verification of this licence information was required to confirm which of the proposed schemes have been built, what the final designs of the schemes entailed, and the dates that they became operational. This verification involved online searches (search engine: www.Google.co.uk) for the ‘name’ of the proposed scheme and the term ‘hydro’. If no relevant links were found within the first two pages of results on the search engine, then the scheme was deemed not operational. If some relevant links were found in the first two pages of search results for a scheme, then these were used to gather meta-data on the scheme, with further focussed online searches used where evidence suggested that a scheme was operational. This process does not necessarily produce an exhaustive list of operational schemes, but it is based on a systematic and transparent search. The search process identified 161 operational small-scale (< 5MW capacity) ROR HEP schemes in England and Wales out of the 452 schemes that were licenced up until 31^st^ March 2014.

### Systematic identification of ROR HEP schemes with spatially and temporally co-located fish monitoring

Once the operational ROR HEP schemes had been identified a proximity analysis was undertaken, in ArcGIS (v. 10.2), to identify which of the operational schemes had fish monitoring surveillance sites located within a 1 km radius. In order to perform this analysis, the locations of all fish monitoring surveillance sites in England and Wales, were extracted from the respective agency’s databases. Only fish sites that were monitored with standardised single or multiple-pass electro-fishing sampling techniques [21–23] were used. A buffer tool was used to classify these features relative to operational HEP schemes, and the output selection set exported to MS excel. A subsequent manual visual check was then performed for each HEP scheme identified as having spatially co-located fish data, using online mapping tools to ensure that the HEP scheme and the fish monitoring site were indeed located on the same river. This step of the analysis included measurement of the approximate channel pathway distance between the HEP scheme and the fish monitoring site, and recording whether the monitoring site is upstream or downstream of the HEP scheme. Finally, the dates of fish monitoring were compared with the dates that the respective HEP scheme became operational, to ensure that the scheme had fish monitoring data available for both the period before and after installation (referred to herein as temporal co-location).

**Fig 1:**
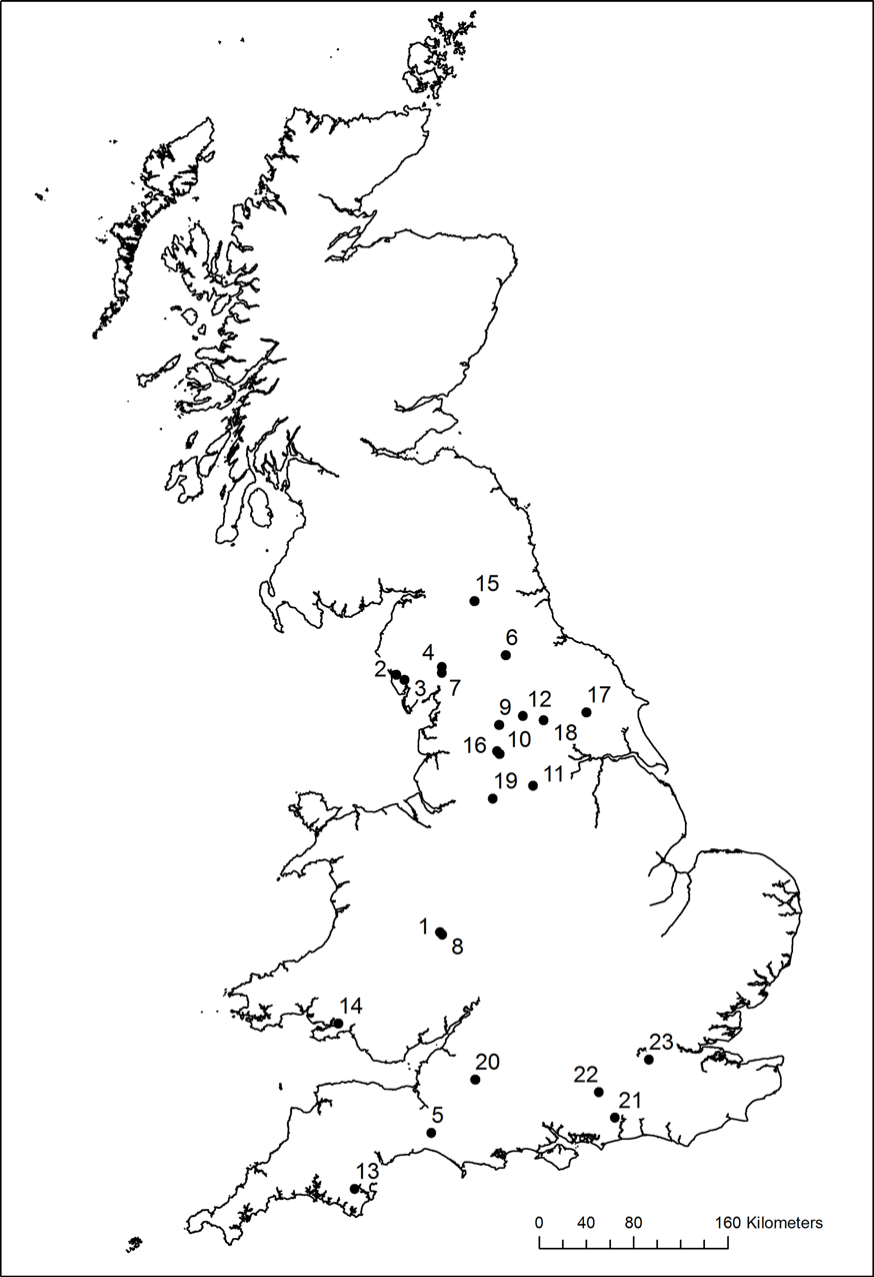
Locations of the 23 HEP schemes with spatially and temporally co-located fish monitoring data. Reprinted from Ordnance Survey (Digimap Licence) under a CC BY license, with permission from Crown Copyright and Database Right [2016].

A total of 23 of the 161 verifiable operational HEP schemes in England and Wales had spatially and temporally co-located fish monitoring surveillance data. As highlighted in S1 Table, the selected ROR HEP schemes incorporate a range of designs (reverse-Archimedes screw turbines, crossflow turbines, Francis turbines, Kaplan turbines, Turgo turbines, waterwheel turbines), power capacities (3–450 kW), and head heights (ranging from low-head schemes that occur in lower gradient river reaches and are retrofitted to existing structures or installed adjacent to existing weirs, to high-head schemes that use relatively small volumes of water from high-gradient, upland rivers, diverted over longer distances. The head in high-head schemes is usually provided by natural waterfalls or cascades, but small weirs are still used to divert water). The 23 schemes are fairly typical of ROR HEP schemes in Europe, the majority of which are mini (<1 MW) and micro (<100 kW) schemes installed on small river systems [12]. As illustrated in Fig 1, the selected ROR HEP schemes also occur across a broad geographic area.

### Systematic identification of control sites with temporally co-located fish monitoring

The fish community response at HEP ‘impact’ sites were compared to those at respective ‘control’ sites that lack the influence of a HEP scheme, but that are: (1) local and therefore likely to have been experienced similar fluctuations in weather and hydrological conditions (floods and droughts) over the corresponding period of monitoring of the ROR HEP ‘impact’ site, and (2) have been influenced by similar historical river management legacies, specifically, the presence of weirs. The second criterion (i.e. presence of weirs) was chosen because most ROR HEP schemes in England and Wales are constructed on, or make use of, one of the 16,822 existing man-made weirs in England and Wales [12, 24], and therefore the fish communities living nearby ROR HEP schemes are unlikely to represent pristine or unaltered communities before the ROR HEP scheme is constructed. For a fairer baseline comparison, the ‘control’ sites should also not start with pristine or unaltered communities [25]. The Environment Agency’s River Obstructions Database [24] provided the location and characteristics of weirs in England and Wales. The control sites were selected using a proximity analysis undertaken in ArcGIS (v. 10.2), to identify weirs within a 20 km radius of each operational ROR HEP scheme. A buffer tool was used to classify these features relative to operational ROR HEP schemes, and the output selection set exported to MS excel.

Once all weirs within 20 km of a HEP scheme had been identified, a secondary proximity analysis was used to identify which of those weirs had fish monitoring surveillance data within a 1 km radius. This proximity analysis used was identical to the process for identifying ROR HEP schemes with spatially and temporally co-located monitoring data (described above), including the subsequent manual visual check. In addition, the periods of fish monitoring at each potential control site were compared with the period of fish monitoring for the respective paired HEP scheme. For most HEP schemes, there were multiple potential control sites identified through the proximity analysis. In order to select a control site for each HEP scheme in a systematic manner, whilst minimising data processing time, the latter manual checks were conducted in an ascending order based on fish monitoring site ID number. Once ten potential sites had been assessed (i.e. ten sites with spatially-co-located monitoring data covering the before and after period of the corresponding HEP scheme), then the process stopped and the fish monitoring site with the greatest number of matched years of monitoring, with regards to the respective HEP scheme’s monitoring, was selected as the control site. For two HEP schemes (schemes 8 and 16) there were no suitable control sites within the initial 20 km radius, and therefore the radius was expanded to 40 km and the proximity analysis and subsequent manual checks were repeated. For one of these schemes (scheme 8), there were still no suitable control sites within 40 km, and therefore the radius was expanded to 80 km and the proximity analysis and subsequent manual checks were repeated.

### Data Analysis

The null hypothesis of the study was that the construction and operation of ROR HEP schemes has no impact on the local (within ~1 km) fish community composition. Six area-normalised metrics of community composition were analysed: fish species richness (the number of species per 100 m^2^), fish abundance (the number of fish, regardless of species, per 100 m^2^), the abundance of Atlantic salmon (*Salmo salar*) per 100 m^2^, the abundance of >1 year old Atlantic salmon per 100 m^2^ (determined from fish length data as those individuals greater than or equal to 100 mm in length), the abundance of brown trout (*Salmo trutta*) per 100 m^2^, and the abundance of >1 year old brown trout per 100 m^2^. The number of species and number of fish metrics were selected to provide an overview of the fish community composition; both of these metrics would generally decline with environmental degradation [26]. The Atlantic salmon and brown trout metrics were selected because these widespread species are an important component of subsistence, recreational and commercial fisheries throughout the European region, and provide a range of ecosystem services [27,28]. Moreover, as anadromous species (though some brown trout belong to lacustrine or resident ecotypes), they may be sensitive to physical barriers to migration and physical degradation of spawning and nursery habitats in rivers—both of which have been attributed to HEP development [27,28]. Furthermore, owing to the importance of these species, coupled with the high catch-efficiency for these species via the electro-fishing technique, the survey data available for them are more consistent and detailed than is available for some other species; for example, the availability of concomitant body length measurements and scale analyses provides a breakdown of the age structure of the populations which can aid interpretation of any observed population changes.

The hypothesis was tested, following the logic of beyond-BACI designs [19–20], by creating a linear mixed effect (LME) model, in the form of:

Response∼BA*CI+(1|Year)+(1|Season)+(1|Site)

The interactions between Before-After and Control-Impact were modelled as fixed factors, while Year, Season and Site (to allow for paired control/treatment sites) were modelled as random effects. In this design, particular interest lies in the interaction (Before-After * Control-Impact), which, if significant, implies that the fish communities of river sites with ROR HEP schemes (the impact group) responded differently to fish communities of river sites without ROR HEP schemes (the control group). The statistical significance of the interactions was tested via an analysis of variance on the fitted models, using F statistics of the lmer function (lme4 library) available in free software (R 3.2.2). The *p*-values were calculated using the lmTest package within this software, with Satterthwaite approximation for degrees of freedom. Effect sizes were calculated using lsmeans, from the lsmeans package within this software. All data were transformed, using natural logarithm (n+1), prior to analysis to correct for zero inflation and non-normality of data.

For the selected impact and control sites, it was found that the fish sampling method varied within sites (over time), and between sites with regards to the number of electrofishing passes that were conducted (typically between 1 and 3 passes). In order to correct for this, and allow consistent comparison, only the first pass data were used in this study. The first pass of a fish survey is unlikely to capture the entire population of each species, but it does enable the greatest number of comparisons within sites (over time) and between sites. In order to understand the capture efficiency of first pass data, the survey data from all 46 fish monitoring sites were collated and where 3-pass survey data were available (number of 3-pass surveys = 153), a comparison was made of the abundance of fish caught in pass 1, relative to the cumulative abundance of fish caught with 3 passes. On average, pass 1 of an electrofishing survey captured 63% of the abundance of fish (excluding minor species) that were captured with 3 passes.

It was also found that the fish recording also varied within sites (over time), and between sites with regards to the recording of ‘minor species’. Minor species, including minnow (*Phoxinus phoxinus*), bullhead (*Cottus gobio*), stone loach (*Barbatula barbatula*), 3-spined stickleback (*Gasterosteus aculeatus*), and gudgeon (*Gobio gobio*), were sometimes recorded in absolute numbers for each pass, but more typically recorded as a logarithmic abundance category (1–9, 10–99, 100–999, 1000–9999) for the entire survey. This reflects both the poor capture efficiency of these species and the fact that rivers often support very large populations of small fish species, which can make catching and recording all individuals a time consuming and impractical process [29]. In order to correct for this inconsistency in recording, minor species were included in the metric of species richness (number of species per 100 m^2^), even if only a logarithmic abundance category was provided for the entire survey. However, minor species were excluded from the metric of fish abundance (number of fish per 100 m^2^). In rare cases, a non-minor species was recorded in the form of a logarithmic abundance category. In these cases, the central number of the abundance range was used in calculating the abundance of fish (e.g. if the abundance of the fish is estimated to be 1–9, the abundance figure used would be 5) for a single pass survey. Where there were multi-pass surveys but the log abundance of a non-minor species was only recorded for the total survey, the central abundance number was divided by the number of runs when estimating the first run abundance of these fish. The fish community data, processed using the above methodology, are available in S2 Table.

Meta-data on the period of monitoring and the number of fish surveys at each of the 23 impact and 23 control sites is displayed in S3 Table. The average distance between the HEP turbines and the fish monitoring sites was 482 metres. The average period of fish monitoring before construction of the 23 HEP schemes was 126 months (i.e. >10 years of baseline monitoring), with an average of 5 fish surveys conducted per site during this period. The average period of monitoring after the construction of HEP schemes was 50 months (i.e. >4 years), with an average of 3 fish surveys conducted per site during this period. The corresponding periods of fish monitoring for the control sites, were similar at 128 months, with an average of 6 surveys conducted per site during the ‘Before’ period, and an average monitoring period of 50 months, with an average of 3 surveys conducted per site during the ‘After’ period.

## Results

**Fig 2:**
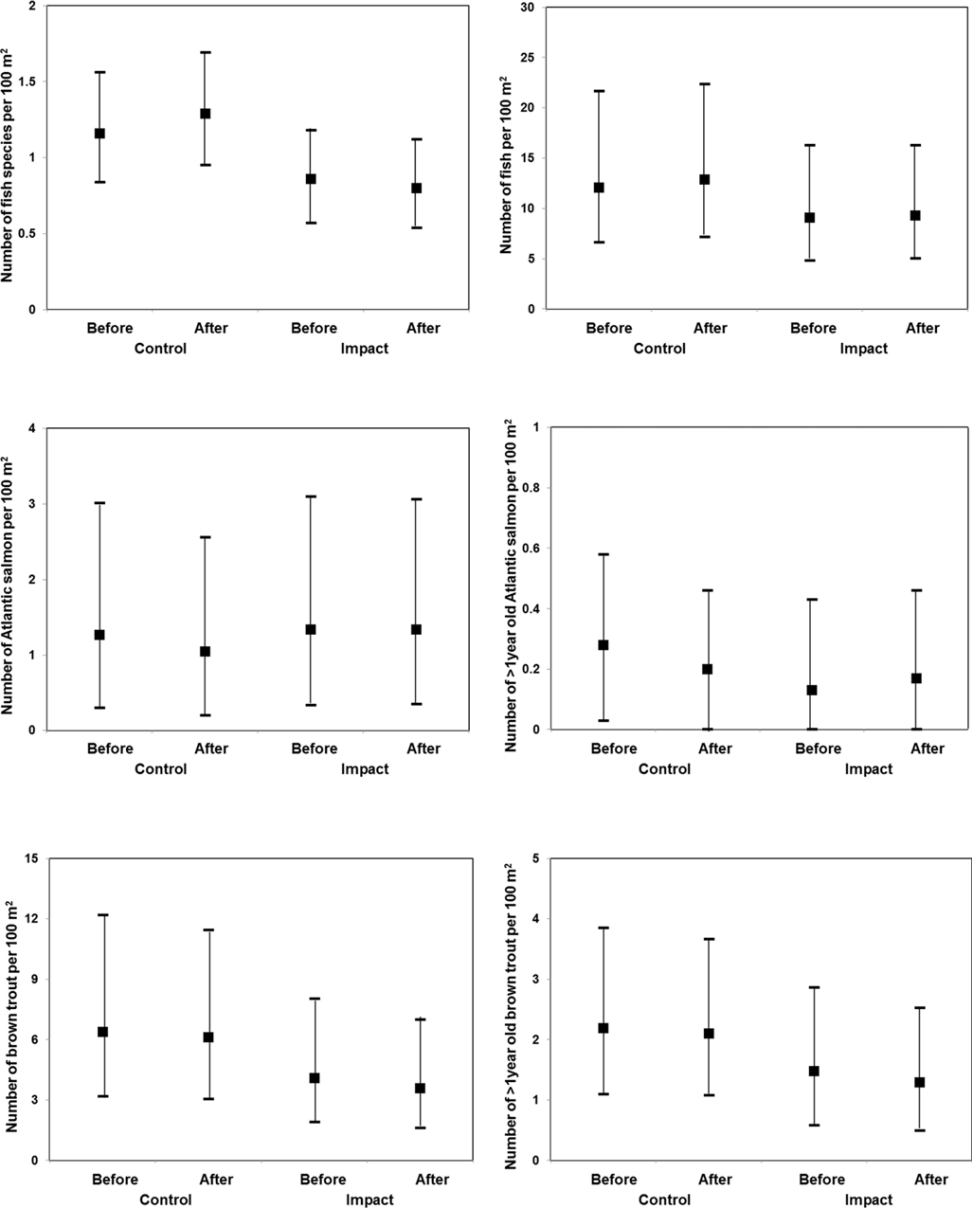
Fitted (least square) mean before and after values for the six area-normalised metrics of fish community composition for control and impact groups. Bars illustrate the upper and lower 95% confidence limits.

Table 1 displays the fitted least squares means, standard errors, degrees of freedom, and 95% confidence limits for each treatment (Control-Impact) and period (Before-After), for the six area-normalised metrics of fish community composition. The fitted least squares means and 95% confidence limits are also illustrated in Fig 2. As can be seen from Fig 2, there are only small changes in the mean values for each metric between the before period and after period in both the control and impact groups. There are also wide ranges for the upper and lower 95% confidence limits reflecting the variability of the metrics between sites within each group and within sites over time. Table 2 displays the BACI model outputs for the six area-normalised metrics of fish community composition. As can be seen from Table 2, there was only one metric of fish community composition (species richness) for which the construction and operation of ROR HEP schemes had a statistically significant effect (*p* <0.05). The fitted least squares mean number of species per 100 m^2^ increasing from 1.16 for the before period (95% CI: 0.84–1.86), to 1.29 for the after period (95% CI: 0.95–1.69) in the control group, but decreasing from 0.86 for the before period (95% CI: 0.57–1.18) to 0.80 for the after period (95% CI: 0.54–1.12) in the impact (ROR HEP) group.

**Table 1:** Least squares (LS) mean, standard error (SE), degrees of freedom (DF) and confidence limits (CL) for each treatment (Control-Impact) and period (Before-After), for the six area-normalised metrics of fish community composition.

Fish community metric (n per 100 m2)	Treatment	Period	LS Mean	SE	DF	Lower CL	Upper CL
**Species richness**	Control	Before	1.16	0.08	48.83	0.84	1.56
		After	1.29	0.08	45.59	0.95	1.69
	Impact	Before	0.86	0.08	50.38	0.57	1.18
		After	0.80	0.08	46.21	0.54	1.12
**Fish abundance**	Control	Before	11.94	0.31	38.84	6.54	21.20
		After	12.87	0.28	35.19	7.25	22.34
	Impact	Before	8.68	0.31	40.60	4.64	15.61
		After	8.97	0.30	35.84	4.93	15.61
**Atlantic salmon abundance**	Control	Before	1.27	0.32	37.43	0.30	3.01
		After	1.05	0.31	35.08	0.20	2.56
	Impact	Before	1.34	0.32	38.68	0.34	3.10
		After	1.34	0.31	35.58	0.35	3.06
**Atlantic salmon (>1 year old) abundance**	Control	Before	0.28	0.12	37.90	0.03	0.58
		After	0.20	0.11	36.26	0.00	0.46
	Impact	Before	0.13	0.13	40.32	0.00	0.43
		After	0.17	0.12	36.77	0.00	0.46
**Brown trout abundance**	Control	Before	6.39	0.32	32.71	3.18	12.20
		After	6.10	0.31	30.92	3.06	11.43
	Impact	Before	4.10	0.32	33.52	1.89	8.03
		After	3.57	0.32	31.25	1.61	7.00
**Brown trout (>1 year old) abundance**	Control	Before	2.19	0.22	23.52	1.10	3.85
		After	2.10	0.22	22.98	1.08	3.66
	Impact	Before	1.48	0.25	29.58	0.58	2.86
		After	1.29	0.23	26.84	0.49	2.53

**Table 2:** BACI model effect size and standard error (in parenthesis) for the six area-normalised metrics of fish community composition.

	Dependent variable					
	Number of species	Number of fish	Number of Atlantic salmon	Number of >1year old Atlantic salmon	Number of brown trout	Number of >1year old brown trout
**B-A**	0.06**	0.07	-0.10	-0.07	-0.04	-0.02
	(0.03)	(0.12)	(0.10)	(0.05)	(0.09)	(0.08)
**C-I**	-0.14	-0.25	0.03	-0.12	-0.31	-0.22
	(0.12)	(0.28)	(0.30)	(0.16)	(0.25)	(0.19)
**BACI**	-0.08**	-0.05	0.10	0.11	-0.07	-0.05
	(0.04)	(0.15)	(0.13)	(0.07)	(0.12)	(0.12)
**Constant**	1.16***	11.94***	1.28***	0.28**	6.42***	2.18***
	(0.08)	(0.30)	(0.31)	(0.11)	(0.32)	(0.22)
**Observations**	382	382	382	304	382	282

Before-after contrast (B-A), control-after contrast (C-I), before-after, control-impact interaction (BACI). Statistical significance

** *p* <0.05

*** *p* <0.01.

The six metrics of fish community composition studied exhibited substantial variability both among sites (see Fig 2) and over time within sites. River sites such as site 6 illustrated in S1 Fig, that show no real long-term trends in their metrics of fish community composition, still nevertheless experienced 4-fold variation in the number of species per 100 m^2^ over time and 8-fold variation in the number of fish per 100 m^2^ over time. Part of this variation is likely to be associated with natural biological cycles and stochastic events, part of the variation may also be associated with the precision of the survey technique. The resultant variability of the metrics influences the power of statistical tests (i.e. probability of correctly detecting a statistically-significant effect when one exists). Statistical power analysis of this study, according to the method described by Stroup [30], revealed that the probability of correctly detecting a statistically significant effect, for the magnitude of effect sizes observed in this study, was 0.62 for the number of species per 100 m^2^, 0.06 for the number of fish per 100 m^2^, 0.12 for number of Atlantic salmon per 100m^2^, 0.31 for the number of Atlantic salmon >1year old per 100 m^2^, 0.09 for the number of brown trout per 100 m^2^, and 0.07 for the number of brown trout >1year old per 100 m^2^.

## Discussion

In this study we investigated the effects of ROR HEP schemes on communities of fish in temperate streams and rivers, using a Before-After, Control-Impact (BACI) study design that is more robust than previous studies [19–20], that makes use of routine environmental surveillance data collected according to standardised methods as part of national and international monitoring programmes. The 23 ROR HEP schemes included in this study were systematically-selected, as were their paired control sites which were located on independent streams/rivers that also had the influence of management legacies (specifically the presence of weirs). The average period of monitoring before construction (>10 years) and after construction (>4 years) in this study is far greater than is normally possible through monitoring commissioned from standard academic research funding or short-term investigative studies.

The BACI effect size estimates are small for all six metrics of fish community composition, with the 95% confidence intervals overlapping zero for five of the metrics. The construction of ROR HEP schemes is estimated to have a small negative effect on the number of species per 100 m^2^ (-0.08, 95% confidence interval -0.158 to -0.002), a small negative effect on the number of fish per 100 m^2^ (-0.05, 95% confidence interval -0.344 to 0.244), a small positive effect on the number of Atlantic salmon per 100 m2 (0.10, 95% confidence interval -0.155 to 0.355), a small positive effect on the number of >1 year old Atlantic salmon per 100 m^2^ (0.11, 95% confidence interval -0.027 to 0.247), a small negative effect on the number of brown trout per 100 m^2^ (-0.07, 95% confidence interval -0.305 to 0.165), and a small negative effect on the number of >1 year old brown trout per 100 m^2^ (-0.05, 95% confidence interval -0.285 to 0.185). However, the results show that there was only one metric of fish community composition (number of species) for which the effect is statistically significant (*p* <0.05). In river sites with ROR HEP schemes, there was a very small decrease in mean species richness, by 0.06 species per 100 m^2^, in the after construction period relative to the before construction period. In control sites there was a small increase in mean species richness, by 0.13 species per 100 m^2^, in the after construction period relative to the before construction period. These results do not suggest that the construction and operation of ROR HEP schemes are causing any catastrophic collapse of the fish community. However, the results could indicate that the construction and operation of ROR HEP schemes could potentially suppress small increases in species richness that may have been observed over time had the ROR HEP schemes been absent. It is worth noting that the control and impact groups were different at baseline with respect to the species richness metric (0.86 species per 100 m^2^ for the impact group; 1.16 species per 100 m^2^ for the control group), and that these differences could have influenced the rates and direction of change from baseline. Nevertheless, these findings warrant further investigation to establish the likely mechanisms of community composition change and to better understand longer-term trends in community composition.

With any inferential statistical test there is always the possibility that when a difference does exist, the test will not be able to identify it. This type of mistake is called a Type II error [31]. The statistical power of a test refers to the probability of making a Type II error. It is generally accepted that statistical power should be 0.8 or greater; that is, studies should have an 80% or greater chance of finding a statistically significant difference when one exists. However, consideration and reporting of statistical power is rare in environmental science studies. A review of fisheries and aquatic science research papers that did not reject some null hypothesis found 98% of the papers failed to report statistical power [31]. Whilst it can be difficult to achieve a power of 0.8 in environmental studies and other disciplines [32], it is better for authors to acknowledge what their power was, rather than to ignore it, because the results of studies with low statistical power can be both misleading and dangerous not only because of their inability to detect ecologically significant changes, but also because they create the illusion that something useful has been done [33]. Statistical power analysis for this study revealed that the probability of correctly detecting a statistically significant effect, for the magnitude of effect sizes observed in this study was 0.62 for the number of species per 100 m^2^, 0.06 for the number of fish per 100 m^2^, 0.12 for number of Atlantic salmon per 100m^2^, 0.31 for the number of Atlantic salmon >1year old per 100 m^2^, 0.09 for the number of brown trout per 100 m^2^, and 0.07 for the number of brown trout >1year old per 100 m^2^. For this study to have had a statistical power of 0.8, which is often regarded as a desirable threshold, the differences in response between the control and impact groups would need to have been 17% bigger than the observed difference for the number of species per 100 m^2^ metric, but almost 200% bigger than the observed difference for the number of fish per 100 m^2^ metric. For prospective studies, the statistical power could be increased through an increased number of passes within the fish surveys at each site and an increased number of fish surveys at each site, in addition to an increased number of sites within the study. The data used here were assembled from public sources with statistical noise introduced from variation in both the sampling methods and effort. By designing sampling with statistical analysis in mind [20], these external effects can be minimized, and sampling effort can be more efficiently allocated. Future research should take this statistical power analysis into consideration when attempting to design studies to detect the impacts of interventions on fish communities in temperate streams and rivers.

Previous research on ROR HEP schemes has reported similar findings to those observed here. For example, a recent non-peer-reviewed report [34] examined before and after construction fish community composition data for 10 high-head ROR HEP schemes (ranging in capacity from 0.68 to 3 MW) in the temperate climatic zone of Scotland. Most of the HEP schemes had fish monitoring data in the depleted reaches (i.e. a section of river with lower flows due to diversion of some of the flow through the HEP turbine) and ‘control’ reaches upstream or downstream of the depleted reaches. This report examined each HEP scheme separately, focussing on potential impacts on populations of Atlantic salmon and brown trout. It concluded that there were no statistical significant differences between fish communities within depleted reaches and upstream and/or downstream ‘control’ sites before and after installation of HEP schemes. A peer-reviewed article by Santos et al [35] reported the results of a post-construction spatial analysis of fish communities upstream and downstream of 18 ROR HEP schemes (ranging in capacity from 0.30 to 8.7 MW) in the Mediterranean climatic zone of central and northern Portugal. This study concluded that, unlike the patterns observed in large storage-type HEP schemes, neither fish species richness nor fish abundance differed significantly between sites upstream and downstream of ROR HEP schemes; though there were statistically-significant differences in the size-structure of fish populations upstream and downstream of the schemes for one species of fish (a greater proportion of smaller individuals downstream) for schemes that the authors classified as having ‘suitable’ fish passes, and significant differences for three species of fish (greater proportion of smaller individuals upstream) for schemes with ‘unsuitable’ fish passes. However, caution should be exercised when interpreting the results of previous research in this field; the studies are often constrained by the absence of long-term standardised data and weak study design; mainly relying on post-construction observations (e.g. Santos et al., [35]), and/or spatial comparison with upstream and/or downstream reference reaches (e.g. [34, 35]), which limits the conclusions that can be drawn [12,18]. One of the reasons for this is that many of the potential impacts of ROR HEP schemes would not be isolated to the hydrologically-depleted reach; therefore non-depleted reaches of rivers, upstream and/or downstream of ROR HEP schemes, would not represent independent control sites. For example, if ROR HEP schemes were responsible for direct fish mortality as a result of contact with turbine blades, or a decline in fish population associated with increased barriers to migration, then we may expect to observe these effects in fish populations upstream and downstream of the hydrologically-depleted reach as well as in the depleted reach itself. When studies lack independent control sites, it is more difficult to detect a change and to ascertain whether any observed change in the fish community was caused by the intervention of interest, or by other factors affecting the sites within the region of interest (e.g. droughts, floods) [25]. Furthermore, if a study only has post-treatment monitoring, but the control and treatment groups were different at baseline (before installation of the ROR HEP scheme), or in the case of the Santos et al [35] study the upstream and downstream stretches comparators were different (e.g. due to pre-existing natural differences in habitat suitability), then the study is vulnerable to making unfair comparisons and drawing incorrect conclusions. Run-of-river HEP schemes, at least those in Europe, tend to be constructed on sites with existing weirs and barriers [12, 24] and therefore they are likely to have different fish communities at baseline relative to pristine sites in similar but more natural environments.

In this study different types of ROR HEP schemes were grouped together, regardless of design features such as turbine type, capacity, or head height. The authors recognise that different scheme designs may have different biological impacts, but we were not able to conduct any sub-analysis owing to the limited number of replicates of each scheme design and the limited statistical power. The effect observed is the average response monitored an average of 482m upstream or downstream from ROR turbines with an average capacity ~54kW. It may be possible to conduct a follow-on BACI study with a sub-analysis for ROR HEP scheme design, if it is possible to add data from further ROR HEP schemes with paired controls that have been monitored in a similar manner by regulatory authorities within other countries. If such a study were conducted, it would be useful to investigate the distances upstream/downstream over which any impacts are observed. This would help gain better constraints on the absolute effects (e.g. number of fish mortalities) in relation to scheme design, but would also enable comparative lifecycle analysis against other sources of electricity (e.g. fish mortality per kW h^-1^ generated). However, it should be recognised that the results may be regulation-specific, and that most of the ROR HEP schemes included in this study have been developed in accordance with best-practice guidance from the respective regulatory authorities of England and Wales [36–38]. This guidance details the regulatory requirements stipulating where/when it is necessary to install a fish pass, to include fish screens, and/or to halt abstraction/operation of the ROR HEP scheme during low flows. Ten of the ROR HEP schemes in this study had fish passes constructed as part of a licence requirement, and two other ROR HEP schemes in the study were constructed on weirs with existing fish passes prior to construction. Furthermore, twenty one of the ROR HEP schemes included in this study had hands-off flow thresholds as part of their licence, to prevent abstraction during low flows. For ROR HEP schemes built in countries with a significantly different set of regulatory requirements, the effects of the schemes may be dissimilar to those observed in this study. The significance of country-specific environmental regulation in determining the potential impacts of ROR HEP schemes is noted by Kubecka et al [39], who based on the findings of a post-construction spatial analysis of fish communities upstream, downstream and within the depleted reaches of 23 ROR HEP schemes (up to 10 MW in capacity; most < 100kW) in the temperate climatic zone of the Czech Republic, suggested that water abstraction caused succession from large-bodied fish species (adult brown trout, chub, dace, grayling) towards small-bodied fish (trout fry, minnow, bullhead, stone loach, gudgeon) within the depleted reaches. Kubecka et al [39] noted that most of these HEP schemes were developed when ‘environmental legislation was not fully developed and most of the stations were operated with little regard for ecological considerations’.

## Supporting Information

S1 Fig Time-series of fish community composition for ROR HEP scheme 6: An example of temporal variability in metrics of fish community composition within a site. (TIF)

S1 Table Meta-data on each HEP scheme. (DOCX)

S2 Table Fish community data for each survey, for each site, for the six metrics of fish community composition. (XLSX)

S3 Table Meta-data on fish monitoring for each impact and control site. Asterisks indicate that fish monitoring is within the depleted reach of a HEP scheme.
(DOCX)
