# Supplementary material for: The Effects of Run-of-River Hydroelectric Power Schemes on Fish Community Composition in Temperate Streams and Rivers
Source: PLoS One. 2016 May 18;11(5):e0154271. doi: 10.1371/journal.pone.0154271 (PMC4871443; doi:10.1371/journal.pone.0154271)
Supplement: S1 Table — (DOCX) [file pone.0154271.s002.docx]

**S1 Table. Meta-data on each HEP scheme**

| **HEP Scheme** | **Turbine Type** | **Capacity (kW)** | **Head (m)** | **Construction Start Date** | **Layout** | **Fish pass constructed as part of development** | **Hands-off flow (Q: m3 s-1)** |
| --- | --- | --- | --- | --- | --- | --- | --- |
|  |  |  |  |  |  |  |  |
| 1 | Kaplan | 165 | 1.5 | May-12 | 5 m depleted reach | Larinier | Q95 |
| 2 | Turgo | 100 | 58.5 | Jan-13 | 730 m depleted reach | Eel pass (brush slope) | Q85: 0.080 |
| 3 | Turgo | 450 | 96 | Jan-10 | 1150 m depleted reach | Alaskan | Q90: 0.053 |
| 4 | Turgo | 15 | 30 | Aug-09 | 200 m depleted reach | None | Q95: 0.012 |
| 5 | Crossflow | 6 | 3 | Jun-06 | 65 m depleted reach | Bespoke | 0.03 |
| 6 | Vane-regulated Propellor | 3 | 3.1 | Oct-06 | 130 m depleted reach | None | Q95: 0.107 |
| 7 | Archimedean Screw | 100 | 3.5 | Feb-13 | 30 m depleted reach | Larinier | Q90: 0.470 |
| 8 | Waterwheel | 3 | 0.9 | Jun-09 | 190 m depleted reach | None | Q90: 1.98 |
| 9 | Crossflow | 15 | 2.7 | Apr-09 | 430 m depleted reach | None | Q95 |
| 10 | Archimedean Screw | 10 | 2.2 | May-12 | 10 m depleted reach | None: Existing fish pass | Q95 |
| 11 | Archimedean Screw | 9 | 3.4 | Jan-09 | 450 m depleted reach | None | Q95 |
| 12 | Waterwheel | 11 | 4.5 | Jun-09 | 295 m depleted reach | None | None |
| 13 | Waterwheel | 10 | 9.7 | Jul-04 | 685m depleted reach | None | 0.015 |
| 14 | Archimedean Screw | 30 | 4.7 | Jul-13 | 15 m depleted reach | Eel pass (brush slope) | Q90: |
| 15 | Francis | 3 | 6.5 | Nov-06 | 15 m depleted reach | None | None |
| 16 | Francis and Crossflow | 10.5 | 4.8 | Mar-05 | 150 m depleted reach | None | Q95 |
| 17 | Archimedean Screw and Waterwheel | 24 | 1.8 | Aug-09 | On weir (Archimedean) and 90 m (Waterwheel) | None | Q95 |
| 18 | Archimedean Screw | 99 | 2.3 | Jul-13 | 15 m depleted reach | Larinier | Q95: 1.3 |
| 19 | Archimedean Screw x 2 | 68 | 1.4 | Apr-12 | 5 m depleted reach | Fish ladder | Q95: 1.57 |
| 20 | Archimedean Screw | 66 | 2.2 | Aug-10 | 220 m depleted reach | None | Q95: 0.587 |
| 21 | Archimedean Screw | 15 | 8.5 | Jan-12 | 5 m depleted reach | None: Existing Alaskan fish pass | Q95:1.079 |
| 22 | Archimedean Screw | 11 | 1.5 | Aug-11 | 5 m depleted reach | Bespoke | Q95: 0.500 |
| 23 | Archimedean Screw | 8.5 | 1.2 | Mar-12 | 15 m depleted reach | Larinier and Eel pass (brush slope) | Q95: 1.130 |
