# Supplementary material for: The Effects of Run-of-River Hydroelectric Power Schemes on Fish Community Composition in Temperate Streams and Rivers
Source: PLoS One. 2016 May 18;11(5):e0154271. doi: 10.1371/journal.pone.0154271 (PMC4871443; doi:10.1371/journal.pone.0154271)
Supplement: S3 Table — Asterisks indicate that fish monitoring is within the depleted reach of a HEP scheme. (DOCX) [file pone.0154271.s004.docx]

**S2 Table. Meta-data on fish monitoring for each impact and control site. Asterisks indicate that fish monitoring is within the depleted reach of a HEP scheme.**

| **Scheme** | **Monitoring site** | **Latitude** | **Longitude** | **Distance (m) of monitoring upstream (US) or downstream (DS) from HEP turbine (or weir for control sites)** | **Period of monitoring before construction started (months)** | **Number of surveys before construction started** | **Period of monitoring after construction started (months)** | **Number of surveys after construction started** |
| --- | --- | --- | --- | --- | --- | --- | --- | --- |
|  |  |  |  |  |  |  |  |  |
|  |  |  |  |  |  |  |  |  |
|  |  |  |  |  |  |  |  |  |
|  |  |  |  |  |  |  |  |  |
| 1 | CONTROL | 52.284627 | -2.8428698 | 280 (US) | 226 | 10 | 27 | 2 |
|  | IMPACT | 52.383722 | -2.7548388 | 85 (DS) | 95 | 6 | 26 | 1 |
| 2 | CONTROL | 54.478776 | -3.522302 | 1000 (US) | 88 | 1 | 18 | 1 |
|  | IMPACT | 54.339006 | -3.3538983 | 760 (DS) | 221 | 3 | 18 | 1 |
| 3 | CONTROL | 54.322034 | -3.0927464 | 300 (DS) | 220 | 12 | 44 | 3 |
|  | IMPACT | 54.301068 | -3.2532709 | 820 (DS)* | 196 | 2 | 19 | 1 |
| 4 | CONTROL | 54.369865 | -2.6822963 | 600 (DS) | 192 | 3 | 60 | 3 |
|  | IMPACT | 54.403066 | -2.7658792 | 585 (DS) | 192 | 2 | 60 | 2 |
| 5 | CONTROL | 50.894789 | -3.0548414 | 85 (DS) | 107 | 6 | 96 | 7 |
|  | IMPACT | 50.853864 | -2.8335736 | 225 (DS) | 107 | 3 | 48 | 1 |
| 6 | CONTROL | 54.624512 | -1.8234052 | 490 (DS) | 49 | 5 | 93 | 6 |
|  | IMPACT | 54.496828 | -1.9307974 | 100 (US) | 131 | 7 | 97 | 7 |
| 7 | CONTROL | 54.215148 | -2.6675822 | 300 (DS) | 209 | 3 | 19 | 1 |
|  | IMPACT | 54.358365 | -2.7641393 | 640 (DS) | 233 | 11 | 17 | 2 |
| 8 | CONTROL | 52.519948 | -1.6447833 | 930 (US) | 163 | 4 | 48 | 1 |
|  | IMPACT | 52.366387 | -2.7249643 | 940 (US) | 216 | 4 | 48 | 1 |
| 9 | CONTROL | 54.04223 | -1.897651 | 215 (DS) | 154 | 12 | 64 | 5 |
|  | IMPACT | 53.964457 | -2.0170962 | 265 (DS) | 20 | 1 | 65 | 2 |
| 10 | CONTROL | 53.682782 | -1.8736013 | 200 (US) | 152 | 10 | 39 | 3 |
|  | IMPACT | 53.742592 | -2.0121983 | 105 (DS) | 56 | 4 | 39 | 3 |
| 11 | CONTROL | 53.321841 | -1.6508499 | 10 (US) | 135 | 5 | 66 | 6 |
|  | IMPACT | 53.501541 | -1.5810077 | 915 (US) | 184 | 2 | 32 | 1 |
| 12 | CONTROL | 54.093124 | -1.7810213 | 80 (DS) | 82 | 5 | 50 | 2 |
|  | IMPACT | 54.033107 | -1.706221 | 1100 (DS) | 82 | 6 | 75 | 5 |
| 13 | CONTROL | 50.414329 | -3.960572 | 590 (DS) | 23 | 3 | 121 | 10 |
|  | IMPACT | 50.416024 | -3.7410532 | 900 (US) | 98 | 3 | 119 | 3 |
| 14 | CONTROL | 51.80977 | -3.978486 | 200 (DS) | 131 | 11 | 13 | 2 |
|  | IMPACT | 51.672502 | -3.9902135 | 530 (DS) | 46 | 1 | 25 | 2 |
| 15 | CONTROL | 54.904723 | -2.2682085 | 150 (DS) | 134 | 2 | 92 | 6 |
|  | IMPACT | 54.906332 | -2.3430799 | 125 (US) | 25 | 1 | 58 | 1 |
| 16 | CONTROL | 54.093498 | -2.0336015 | 750 (US) | 103 | 7 | 113 | 3 |
|  | IMPACT | 53.765441 | -2.0430458 | 130 (DS) | 55 | 5 | 125 | 11 |
| 17 | CONTROL | 53.890207 | -0.8435863 | 40 (US) | 85 | 1 | 34 | 1 |
|  | IMPACT | 54.055996 | -0.88649756 | 5 (US) | 95 | 7 | 71 | 3 |
| 18 | CONTROL | 53.906045 | -1.6483595 | 10 (US) | 204 | 15 | 11 | 1 |
|  | IMPACT | 53.998047 | -1.4403817 | 1300 (US) | 131 | 10 | 23 | 2 |
| 19 | CONTROL | 53.503154 | -2.1235414 | 350 (DS) | 128 | 6 | 30 | 2 |
|  | IMPACT | 53.401753 | -2.0971669 | 200 (DS) | 214 | 6 | 26 | 1 |
| 20 | CONTROL | 51.288381 | -2.2012819 | 180 (US) | 83 | 4 | 22 | 1 |
|  | IMPACT | 51.263338 | -2.3056532 | 660 (US) | 83 | 5 | 37 | 2 |
| 21 | CONTROL | 51.005914 | -0.40364581 | 385 (US) | 102 | 4 | 19 | 1 |
|  | IMPACT | 50.965959 | -0.61732565 | 450 (DS) | 114 | 10 | 42 | 4 |
| 22 | CONTROL | 51.19242 | -0.75924894 | 110 (DS) | 81 | 5 | 38 | 4 |
|  | IMPACT | 51.162127 | -0.80526827 | 175 (DS) | 99 | 7 | 34 | 1 |
| 23 | CONTROL | 51.453054 | -0.3923879 | 990 (DS) | 82 | 3 | 42 | 4 |
|  | IMPACT | 51.402138 | -0.18767975 | 75 (DS) | 216 | 5 | 39 | 2 |
